# Curcumin as a Dietary Additive in Early-Finished Feedlot Steers and Its Effects on Performance, Ruminal Environment, Animal Health, and Meat Quality

**DOI:** 10.3390/ani16020174

**Published:** 2026-01-07

**Authors:** Maisa Damo, João Gustavo Weschenfelder Wandscheer, Mateus Henrique Signor, Charles Marcon, Luisa Nora, Ana Carolina Hadlich Xavier, Roger Wagner, Marcelo Vedovatto, Aleksandro Schafer da Silva

**Affiliations:** 1 Departamento de Zootecnia, Universidade do Estado de Santa Catarina, Chapecó 89815-630, SC, Brazil; maisa.d@edu.udesc.br (M.D.); jgw.wandscheer@edu.udesc.br (J.G.W.W.); 2 Programa de Pósgraduação em Zootecnia, Universidade do Estado de Santa Catarina, Chapecó 89815-630, SC, Brazil; mateus.signor@edu.udesc.br (M.H.S.); charles.giacomelli0864@edu.udesc.br (C.M.); 3 Programa de Bioquímica e Biologia Molecular, Universidade do Estado de Santa Catarina, Lages 88508-500, SC, Brazil; luisa.nora@edu.udesc.br; 4 Deparamento de Ciências de Alimentos, Universidade Federal de Santa Maria, Santa Maria 97105-900, RS, Brazil; anacarolinahadlich5@gmail.com (A.C.H.X.); rogerwag@gmail.com (R.W.); 5 Beef Cattle Nutrition, Louisiana State University, Baton Rouge, LA 70803, USA; mv.vedovatto@gmail.com

**Keywords:** curcuminoids, performance, antioxidants, immunomodulator

## Abstract

The present study aimed to evaluate whether the inclusion of curcumin as a performance enhancer in finishing cattle has positive effects on animal production, the ruminal environment, hematological and biochemical markers, as well as meat quality. Sixteen castrated Holstein steers, 8 months of age, with an average body weight of 247 ± 3.89 kg were divided into two groups: Control, consisting of animals that consumed 144 mg of monensin/animal/day (n = 8); Treatment, consisting of animals that consumed 552 mg of curcumin/animal/day (n = 8). Animals were fed a diet containing 44% roughage (corn silage) and 56% concentrate for 105 days. During this period, body weight measurements were recorded, and blood samples were collected for the determination of hematological, biochemical, and immunological variables. At the end of the experimental period, animals were slaughtered and meat samples were collected for evaluation. No differences were observed between groups for animal performance, feed intake, or feed efficiency. A lower leukocyte count (lymphocyte and granulocyte) and a higher platelet count were observed in animals that consumed curcumin. These animals also exhibited higher cholesterol levels, along with lower circulating glucose concentrations compared to the control group. In the ruminal environment, higher bacterial activity and greater protozoal counts were observed in the treatment group, but no effects on ruminal short-chain fatty acids were observed. Higher activity of the enzymes glutathione S-transferase in serum and superoxide dismutase in meat was observed, combined with lower lipid peroxidation in serum and meat. Meat from steers fed curcumin showed greater yellow color intensity, higher water-holding capacity, and a higher proportion of monounsaturated fatty acids compared to the control group. These results suggest that curcumin can be used as a performance enhancer, similar to monensin, when thinking about performance, but beyond that, curcumin triggered anti-inflammatory and antioxidant action.

## Simple Summary

Curcumin, when consumed by ruminants, has potential anti-inflammatory, antioxidant, and antimicrobial properties. Beef cattle that consumed curcumin in feedlots had lower lymphocyte counts, as well as elevated antioxidant enzymes, combined with reduced lipid peroxidation. Rumen fluid showed higher protozoan counts and greater bacterial activity when curcumin was ingested. Greater water retention capacity in meat, combined with less oxidation, was observed in animals that consumed curcumin. A higher quantity of monounsaturated fatty acids was observed in the meat when curcumin was consumed.

## 1. Introduction

Curcumin is a phenolic compound extracted from *Curcuma longa* L., a rhizome popularly known as turmeric (Açafrão-da-índia) [1]. Turmeric extract is widely used in cooking due to its intense yellow pigment and aroma; however, its main scientific relevance lies in its biological activities, including anti-inflammatory, antioxidant, antimicrobial, antiviral, and immunomodulatory effects [2].

The anti-inflammatory and antioxidant actions of curcumin result from the inhibition of several molecules involved in the arachidonic acid cascade (inflammatory cascade), including lipoxygenases (LOX) and cyclooxygenase-2 (COX-2) [3]. The arachidonic acid cascade occurs through two main pathways. The LOX pathway involves the action of 5-lipoxygenase (5-LOX) on arachidonic acid via oxidation, generating leukotrienes (LTs), which play an important role in the development and persistence of inflammatory responses, as well as monohydroperoxyeicosatetraenoic acids and hydroxyeicosatetraenoic acids, which are associated with pro-inflammatory activity [4]. The cyclooxygenase (COX) pathway is responsible for the first two steps of prostanoid synthesis, molecules that act as important inflammatory mediators [5] and are also involved in the scavenging of reactive oxygen species under conditions of oxidative imbalance [6]. It is important to emphasize that the anti-inflammatory effect of additives is desirable in production animals, since the inflammatory process requires a large amount of ATP, energy that could be used to increase performance and improve meat quality [7].

Studies have shown that the use of curcumin as a dietary additive in nursing lambs improves growth performance, immune system responses, and antioxidant status [8,9,10]. Similar effects have been observed in dairy calves, in which curcumin fed resulted in increased weight gain [11], along with anti-inflammatory and antioxidant effects [12]. In beef cattle, one of the few studies in which curcumin was used as the sole additive demonstrated that curcumin intake altered ruminal microbiota (increasing bacterial populations and reducing protozoa), decreased ruminal ammonia concentration, reduced fecal nitrogen excretion, and increased nitrogen retention [13]. More commonly, studies have evaluated curcumin in combination with other additives in beef cattle diets, reporting positive effects on animal performance [14,15].

The curcumin modulates ruminal microbiota [13] in a manner similar to monensin [16]; we hypothesized that curcumin could act as a performance enhancer in feedlot cattle. But very little is still known about the ruminal effect of curcumin, unlike what is already known about monensin, the main performance enhancer for ruminants. Monensin acts primarily against Gram-positive bacteria, promoting important changes in the end products of fermentation by increasing propionate, which is a direct precursor of hepatic glucose, thus increasing energy efficiency and consequently improving performance [17]. Therefore, the objective of the present study was to evaluate whether the inclusion of curcumin in the diet of finishing cattle has positive effects on performance, the ruminal environment, animal health, and meat quality.

## 2. Materials and Methods

### 2.1. Additives

Curcumin was purchased from Shaanxi Jiahe Phytochem Ltd. (Xi’an, China), derived from dried extract of *Curcuma longa* (turmeric). The manufacturer guarantees at least 65% of purity. The purity of curcumin was analyzed using high-performance liquid chromatography (HPLC) following the methodology described by Marcon et al. [18], with a purity of 71.7% of curcumin being verified. Other curcuminoid components such as demethoxycurcumin and bisdemethoxycurcumin were identified at low levels (1.21% and 1.07%, respectively). Monensin was purchased from the company Elanco Animal Health (Rumensin^®^, Greenfield, IN, USA).

### 2.2. Animals and Facilities

The study was conducted in the ruminant sector of the Experimental Farm of the Centro de Educação Superior do Oeste (FECEO), Universidade do Estado de Santa Catarina (UDESC), located in the municipality of Guatambu, Santa Catarina, Brazil. The experimental design included 16 castrated Holstein steers, aged 8 to 12 months, with an average body weight of 247 kg. Animals were divided into two groups and housed in individual pens equipped with feeders and drinkers.

### 2.3. Experimental Design and Diet

The 16 animals were divided into two groups, with 8 animals per treatment. The control group received the main performance-enhancing additive commonly used in feedlot systems, monensin, at a concentration of 25 mg/kg of concentrate. The treatment group received curcumin at a dose of 100 mg/kg of concentrate on a dry matter basis. The curcumin dosage was determined based on a pilot study (unpublished data). The experimental period lasted 105 days.

Diets were formulated according to the nutritional requirements described in BR-Corte 2016 [19], using a concentrate-to-roughage ratio of 60:40, targeting an average daily gain of 1.25 kg. The formulated diet was offered twice daily at 8:00 and 16:30 h; and consisted of corn silage, ground corn, soybean meal, soybean hulls, wheat bran, and a vitamin–mineral premix (Bovigold^®^, Tortuga Agrovet Ltd.a., Recife, Pernambuco, Brazil). Water was provided ad libitum throughout the experimental period.

### 2.4. Animal Performance

Performed in the morning, before feeding the cattle, these were weighed after fasting on days 1, 45, 75, and 105 using a digital scale (Digitron^®^, Digitron da Amazônia Indústria e Comércio Ltd.a, Manaus, Brazil) to obtain body weight values and to calculate weight gain (WG; final body weight—initial body weight) and average daily gain (ADG; WG/number of days between weighings). Feed intake was determined by weighing the diet offered and subtracting refusals, which were weighed the following morning before the first feeding. Based on daily dry matter intake (DMI) and ADG, feed efficiency and feed conversion were calculated. In addition, carcass yield was determined by calculating the percentage of carcass weight relative to live weight prior to slaughter.

### 2.5. Sample Collection

Performed in the morning, before feeding the cattle. Blood samples were collected on days 1, 45, 75, and 105 of the experimental periods via the coccygeal vein. Blood was collected into two vacuum tubes (Vacuoplast^®^, Vacuoplast Indústria e Comércio Ltd.a, São Paulo, Brazil): one without anticoagulant for serum collection and one containing anticoagulant (EDTA) for hematological analyses. After collection, tubes were stored under refrigeration at temperatures below 5 °C until arrival at the laboratory for analysis.

Tubes without anticoagulant were centrifuged at 700 rpm for 10 min to separate serum, which was transferred to microtubes (Eppendorf^®^, Eppendorf AG, Hamburg, Germany) and stored at −20 °C until analysis. Samples collected in EDTA tubes for hematology were analyzed immediately upon arrival at the laboratory.

### 2.6. Feed Analyses

Diet samples were pre-dried in a forced-air oven at 55 °C for 72 h and subsequently ground in a Wiley-type mill (Marconi, model MA340, Marconi Equipamentos Científicos Ltd.a., Piracicaba, Brazil) using a 1-mm mesh sieve. Dry matter content was determined by oven-drying ground samples at 105 °C, and ash content was determined by incineration in a muffle furnace at 600 °C for 12 h [20].

Crude protein content was determined using the micro-Kjeldahl method (Method 984.13) [21]. Neutral detergent fiber (NDF) was determined by placing samples in polyester bags [22] and treating them with neutral detergent solution in an autoclave at 110 °C for 40 min [23]. Acid detergent fiber (ADF) concentrations were determined according to Method 973.18 [21]. Ether extract was determined using an automated fat extraction system. Results in Table 1.

### 2.7. Hematology

Hematological variables were obtained using an EquipVet3000^®^ (Equip Diagnóstica, Itatiba, SP, Brazil) hematological analyzer, which provided erythrocyte, leukocyte, and platelet counts, as well as hemoglobin concentration and hematocrit percentage. Leukocyte differential counts (lymphocytes, granulocytes, and monocytes) were also determined.

### 2.8. Serum Biochemistry

Serum biochemical variables (total protein, albumin, cholesterol, glucose, and urea) were analyzed using commercial kits (Analisa^®^, Gold Analisa Diagnóstica Ltd.a, Belo Horizonte, Brazil) and a semi-automatic analyzer (Bio Plus 2000^®^, Barueri, SP, Brazil). Globulin concentration was calculated mathematically as the difference between total protein and albumin.

### 2.9. Oxidative Status

Lipid peroxidation was assessed by measuring thiobarbituric acid reactive substances (TBARS) according to the method described by Jentzsch et al. [24]. Absorbance was read at 535 nm using a spectrophotometer, and results were expressed as nmol of malondialdehyde per mL of serum. Reactive oxygen species (ROS) levels were determined based on the method described by Ali et al. [25]. Samples were diluted 1:10 with 10 nM Tris buffer (pH 7.4) and incubated with 5 µL of dichlorofluorescein diacetate (DCFH-DA), with fluorescence measured spectrophotometrically at 525 nm. ROS formation was quantified using a standard curve of dichlorofluorescein (DCF) in methanol (0.05–1.0 µM), as described by Ali et al. [25]. Non-enzymatic antioxidant levels in serum (non-protein thiols) were evaluated using the method described by Sedlak and Lindsay [26]. Serum glutathione S-transferase (GST) activity was measured according to the method described by Habig et al. [27].

### 2.10. Ruminal Fluid

Ruminal fluid samples were collected on days 75 and 105 of the experimental period; two hours after the cattle were fed. Ruminal pH was measured using a portable digital pH meter (Testo 205^®^, Testo SE & Co. KGaA, Lenzkirch, Germany). Bacterial activity was assessed using the methylene blue reduction test (MBRT) [28], and protozoa were quantified according to Dehority [29], as described in detail by Molosse et al. [30].

The rumen fluid and feed samples were thawed to 5 °C and manually agitated in order to homogenize them. 1 mL aliquots of the supernatant from rumen fluid samples were collected in polypropylene microtubes (2 mL) and then centrifuged for 5 min (12,300× *g*). After that, 250 μL of the supernatant was removed and transferred to a new microtube containing 250 μL of formic acid. The mixture was manually shaken and centrifuged again for 3 min. Then, 250 μL of the supernatant of the mixture was collected into an injection vial and added of 500 μL of 3-octanol solution (665 μg mL^−1^ in methanol) used as an internal standard, and homogenized. The samples were analyzed in a gas chromatograph equipped with a flame ionization detector (GC-FID^®^; Varian Star 3600, Varian Inc., Palo Alto, CA, USA) and an autosampler (Varian 8200CX, Varian Inc., Palo Alto, CA, USA). 1 μL of the extract was injected in split mode at 1:10. The carrier gas used was hydrogen at a constant pressure of 20 psi. The analytes (acetic, propionic, butyric, valeric, and isovaleric acids) were separated by a CP-WAX 52CB capillary column (60 m × 0.25 mm; 0.25 μm stationary phase thickness). The initial column temperature was set at 80 °C for 1 min and increasing to 120 °C at a rate of 8 °C min^−1^, then up to 230 °C by 15 °C min^−1^, where it remained for 1 min. Injector and detector temperatures were set at 250 °C. The validation of the method comprised the following parameters: selectivity, linearity, linear range, repeatability, precision, limit of detection (LOD) and limit of quantification (LOQ) for acetic, propionic, butyric, valeric and isovaleric acids. Analytical parameters are shown in Table S1. Linearity was evaluated by calculating a regression equation using the least squares method. LOD and LOQ values were achieved by sequential dilutions up to signal-to-noise ratios of 3:1 and 6:1, respectively. Precision was assessed by analyzing the repeatability of six replicate samples. Accuracy was determined by recovering known amounts of standard substances added to a diluted sample. The results were expressed in mmol L^−1^ of each SCFA in rumen fluid.

### 2.11. Post-Mortem Evaluations

After slaughter, samples of the longissimus thoracis muscle were collected from all animals, stored under refrigeration at 6 °C for 24 h wrapped in cling film, and used for the determination of meat color, pH, and water-holding capacity (WHC). Color was evaluated on the meat surface against a white background using a CR-400 Chromameter (Minolta, Osaka, Japan; CIE1976 Lab* and CIE L* C* h*), with a standard CIE 1931 illuminant (x_2_λ, yλ, zλ) and a standard calibration plate (No. 1849-701). The L* coordinate represents lightness, whereas a* and b* represent chromaticity coordinates, with a* indicating red–green variation and b* indicating yellow–blue variation.

Thirty minutes prior to color evaluation, sample surfaces were exposed to air to allow myoglobin oxygenation. Three color measurements were taken at random positions on each sample. Meat pH was determined using a portable penetration electrode (HANNA, HI 99163, Hanna Instruments, Inc., Woonsocket, RI, USA). Water-holding capacity was determined by compression using the method of Hamm, adapted by Yamamoto et al. [31].

### 2.12. Statistical Analyses

All data were analyzed using the MIXED procedure of SAS (SAS Inst. Inc., Cary, NC, USA; version 9.4), with the Satterthwaite approximation used to determine denominator degrees of freedom for tests of fixed effects. Body weight gain, feed intake, and feed efficiency were analyzed for the fixed effect of treatment, using animal (treatment) as a random effect. Body weight and all blood variables were analyzed as repeated measures and tested for the fixed effects of treatment, day, and treatment × day interaction, using animal (treatment) as a random effect. Day 1 values (body weight and blood variables) were included as an independent covariate. But to generate treatment means, day 1 data were removed from the dataset but retained as a covariate. A first-order autoregressive covariance structure was selected based on the lowest Akaike information criterion. Means were compared using the PDIFF method, and all results were reported as least squares means (LSMEANS) followed by the standard error of the mean (SEM). Statistical significance was declared at *p* ≤ 0.05.

## 3. Results

### 3.1. Animal Performance

Regarding animal performance variables, no significant differences were observed between treatments (*p* > 0.05), as shown in Table 2. The average daily gain of cattle consuming curcumin was 1.25 kg, which was exactly the value projected during diet formulation, whereas the average daily gain of cattle in the control group was slightly below the formulated target (1.22 kg). Cattle in both groups consumed more than 9 kg of dry matter per day, which allowed the daily intake of additives to be calculated as 144 mg of monensin/animal/day for the control group; and 552 mg of curcumin/animal/day for the treatment group.

### 3.2. Complete Blood Count

Hematological variables were presented in Table 3. No difference was observed between groups for the red blood cell series, i.e., erythrocyte count, hemoglobin, and hematocrit (*p* > 0.05). However, the total leukocyte, lymphocyte, and granulocyte counts were lower in animals that consumed curcumin compared to the control, showing a treatment effect and a treatment x day interaction. The number of monocytes did not differ between treatments (*p* > 0.05). The number of platelets was higher in animals in the curcumin treatment group on days 45 and 75 compared to the control.

### 3.3. Serum Biochemistry

The serum biochemistry results are presented in Table 4. No treatment effect or treatment × day interaction was observed for the variables albumin, globulin, total protein, and urea (*p* > 0.05). However, lower glucose concentrations were observed in cattle that consumed curcumin on days 75 and 105 compared to the control group. Simultaneously, during the same period, cholesterol levels were higher in the steers in the curcumin treatment group.

### 3.4. Oxidative Status

Table 5 shows the results regarding the variables related to oxidative status. Lipid peroxidation is expressed as TBARS, which was lower in the treatment group in all analysis periods (d 45, 75 and 105) compared to the control. In the same periods, we observed higher levels of total thiols in the serum of cattle in the treatment group. GST enzyme activity showed only a treatment effect, being higher in the serum of cattle that consumed the additive compared to the control.

### 3.5. Ruminal Liquid

Results of rumen fluid parameters were presented in Table 6. Higher bacterial activity (↓ MBRT) and also a greater number of protozoa were observed in the rumen fluid of cattle that consumed curcumin compared to the control. However, no treatment effect or treatment x day interaction was observed for pH and volatile fatty acids, when added together or investigated individually (Table 6).

### 3.6. Post-Mortem

Meat results were presented in Table 7 and Table 8. There was no effect of the treatment on carcass yield, as well as on meat variables such as luminosity, red color of the meat, moisture percentage, and protein and fat percentage. However, water retention capacity, yellow color, and SOD activity were higher in the meat of cattle that consumed curcumin compared to the control. TBARS levels in the meat of cattle that consumed the curcumin additive were lower, an indicator of lower lipid peroxidation.

Table 8 shows the effect of treatment on fatty acids in the meat, including a lower proportion of myristic acid (C14:0), palmitic acid (C16:0), linolenic acid (C18:3n6), and cis-5,8,11,14,17-Eicosapentaenoic acid (C20:5n3) in the meat of cattle that consumed curcumin. The meat of steers fed curcumin was found in higher proportions of C18:1n9c (oleic acid) and C22:1n9 (erucic acid). The sum of saturated fatty acids was lower and the sum of monounsaturated fatty acids was higher in the meat of steers fed curcumin compared to the control group.

## 4. Discussion

The inclusion of curcumin in the concentrate of early-finished feedlot steers acted as a performance enhancer, as weight gain and feed efficiency were similar to those observed in control animals that consumed monensin, an additive widely used in feedlot systems [32], and therefore adopted as the control treatment in this experiment. To our knowledge, there are no published studies evaluating the effects of curcumin on performance in adult beef cattle; however, experiments with nursing and weaned calves receiving 200 mg/animal/day of curcumin reported expressive weight gain under ad libitum feeding conditions [11]. In contrast, when curcumin was added to the diet of female dairy calves under restricted feeding conditions at a dose of 65.1 mg/kg of dry matter (DM), no effect on weight gain was observed [12].

Most of the ruminal fluid variables evaluated were not affected by curcumin intake; however, two parameters showed marked changes, namely increased bacterial activity and a greater number of protozoa in the rumen. Several studies have demonstrated the antiparasitic and anticoccidial effects of curcuminoids [11,33], as well as effects on ruminal bacterial activity combined with changes in protozoal populations [9]. The significant increase in ruminal protozoa may be associated with differential effects of curcumin. Protozoa play an important role in stabilizing ruminal fermentation, improving nutrient utilization, and enhancing nitrogen recycling [34]. The lower protozoal counts observed in the rumen of control animals may be related to the presence of monensin, which has been consistently reported to reduce ruminal protozoa populations [35].

No significant differences were observed in the proportion of volatile fatty acids in the rumen, suggesting that curcumin and monensin may act through different mechanisms while producing similar outcomes in terms of ruminal fatty acid profiles in feedlot cattle. It is well documented that monensin intake reduces acetate and increases propionate production, thereby improving energetic efficiency and reducing methane production [36,37]. In cattle [13] and sheep [9] that consumed curcumin, there is no classic pattern of effect on acetate and propionate; but both studies show modulation of the ruminal microbiota.

Red blood cell parameters were not affected by curcumin intake, consistent with previous findings in dairy ewes supplemented with 80 mg of curcumin per kg of concentrate [38]. In contrast, white blood cell parameters were affected, with a reduction in leukocyte counts observed in curcumin-fed animals. According to the literature, curcumin exerts immunomodulatory effects on leukocytes. Studies in lambs fed curcumin at 200 mg/kg of concentrate [8], and in dairy ewes receiving 80 mg/kg [38] also reported lower total leukocyte counts, primarily due to reduced lymphocyte numbers compared with control animals. In the present study, in addition to lower lymphocyte counts, beef cattle also exhibited reduced granulocyte counts.

The reduction in these cell populations is associated with the anti-inflammatory and antioxidant properties of curcumin. Lymphocytes are responsible for mounting specific immune responses, which increase metabolic activity and energy expenditure associated with immune system activation [39], ultimately impairing productive performance [40]. Likewise, reduced granulocyte counts are associated with lower energy expenditure, and decreased generation of free radicals during phagocytosis, which is desirable in production animals where weight gain is a primary objective. Changes in lymphocyte counts were expected to be accompanied by alterations in serum globulin concentrations; however, this was not observed here, in contrast to findings reported in other studies [41,42]. The difference between studies may be related to the age of the animals, since in our study we evaluated animals that already had a mature immune response; unlike these other studies.

A significant increase in platelet counts was observed in cattle fed curcumin, which may be interpreted as a positive effect, given the role of platelets in hemostasis through the formation of a platelet plug that prevents excessive blood loss. Platelets are cytoplasmic fragments derived from megakaryocytes differentiated in the bone marrow [43], a process that may be stimulated by the antioxidant protection provided by curcumin to bone marrow cells [44].

The inclusion of curcumin in cattle diets resulted in increased activity of glutathione S-transferase (GST), and higher total thiol levels, which are important antioxidant markers involved in maintaining oxidative balance and preventing or minimizing physiologic oxidative stress. According to the literature, GST catalyzes the conjugation of electrophilic compounds with glutathione (GSH), a non-protein cellular thiol, rendering them more hydrophilic and facilitating their elimination from cells [45,46]. Electrophilic compounds are generated during xenobiotic biotransformation and may undergo reduction reactions leading to free radical formation [47]. Therefore, increased thiol levels combined with enhanced GST activity act synergistically to reduce cellular oxidative stress, resulting in lower TBARS concentrations in curcumin-fed animals, indicating reduced lipid oxidation and peroxidation.

Lower circulating glucose levels were observed in animals fed curcumin; however, the mechanisms underlying this effect remain unclear and warrant further investigation. Nevertheless, reduced blood glucose concentrations may represent a beneficial effect of curcumin, as this compound improves cellular metabolic efficiency, protects mitochondria, and facilitates intracellular glucose utilization for ATP production [48,49]. Serum cholesterol levels were higher in cattle consuming curcumin, an unexpected result, as previous studies in lambs reported reduced cholesterol levels following curcumin fed [50]. Despite increased circulating cholesterol, no effects of curcumin were observed on total lipid content in beef, in contrast to findings reported by Marcon et al. [51], who observed reduced fat content in the meat of curcumin-fed lambs.

An increase in the b* color coordinate (yellowness) of the meat was observed in animals fed curcumin, which may be related to the yellow pigment of curcumin and may be associated to deposition in muscle tissue. Previous studies have reported increased yolk yellowness in eggs from chickens fed curcumin-fed diets [52]. The higher water-holding capacity observed in meat from curcumin-fed cattle has also been reported in lambs [18]. We hypothesize that this positive effect on water-holding capacity is directly related to the antioxidant action of curcumin in meat, as evidenced in the present study by increased activity of the antioxidant enzyme superoxide dismutase combined with reduced lipid peroxidation. These findings indicate that curcumin consumption improved meat quality and may extend shelf life by reducing oxidative reactions.

Curcumin has been shown to modulate fatty acid profiles in sheep milk [38], quail eggs [53], and lamb meat [51]. In the present study, beef from curcumin-fed cattle exhibited a lower proportion of saturated fatty acids, consistent with findings in lamb meat [51]. A higher proportion of monounsaturated fatty acids was observed in beef, differing from lamb meat, in which increases in polyunsaturated fatty acids were reported [51]. The reduction in palmitic acid observed in beef had previously been described in lambs [51], as well as the increased proportion of oleic acid, which has also been reported in milk from curcumin-supplemented ewes [38]. The increase in erucic acid (C22:1n-9) observed in meat from curcumin-fed cattle may be attributed to alterations in ruminal biohydrogenation, leading to greater intestinal flow of oleic acid (C18:1n-9), combined with curcumin-induced modulation of lipid metabolism pathways [54]. Additionally, the antioxidant and anti-inflammatory properties of curcumin may reduce lipid oxidation, favoring the deposition of long-chain monounsaturated fatty acids in muscle tissue.

## 5. Conclusions

The inclusion of 552 mg of curcumin per animal per day in the diet of finishing cattle resulted in productive performance similar to that of control animals receiving monensin. Curcumin intake promoted anti-inflammatory and antioxidant responses, which positively influenced meat quality. A reduction in saturated fatty acids and an increase in monounsaturated fatty acids were observed in the meat of cattle fed curcumin. These findings support the use of curcumin as a potential alternative to monensin in feedlot cattle diets.

## Figures and Tables

**Table 1:** Chemical composition of the total diet fed to cattle.

Variables	Concentrate: Control (%)	Concentrate: Curcumin (%)	Corn Silage (%)
Dry matter	87.89	87.94	30.92
Crude protein	15.47	15.28	8.55
Ash	0.20	0.21	0.11
Ether extract	2.33	2.36	2.38
Neutral detergent fiber	28.80	27.20	44.25
Acid detergent fiber	11.51	12.01	21.9

**Table 2:** Growth performance of cattle consuming of curcumin with additive.

Items	Treatments ^1^		SEM	*p*-Values ^2^	
	Control	Curcumin		Treat	Treat × Day
Body weight (kg)				0.58	0.46
d 1	253.3	240.7	3.96		
d 45	306.3	293.3	3.95		
d 75	356.0	349.0	3.90		
d 105	381.7	372.6	3.81		
Weight gain (kg)	128.3	131.8	1.04	0.30	-
ADG (kg)	1.22	1.25	0.03	0.20	-
Feed intake (kg DM/day)	9.59	9.21	0.75	0.87	0.79
Feed efficiency (kg/kg)	0.127	0.135	0.01	0.56	-

^1^ Treatments were: Control (144 mg monensin/animal/day); and Treatment (552 mg curcumin/animal/day). ^2^ No difference between groups (*p* > 0.05), for treatment effect and treatment × day interaction. SEM: standard error. Not analyzed (-).

**Table 3:** Hemogram of cattle consuming of curcumin with additive.

Items	Treatments ^1^		SEM	*p*-Values	
	Control	Curcumin		Treat ^2^	Treat × Day ^3^
Erythrocytes (×10^6^ µL)				0.26	0.31
d 1	6.97	7.14	0.18		
d 45	8.47	7.77	0.18		
d 75	8.33	8.15	0.17		
d 105	8.46	8.02	0.18		
Average ^2^	8.42	7.98	0.14		
Hematocrit (%)				0.62	0.52
d 1	29.2	29.4	1.41		
d 45	35.6	33.2	1.41		
d 75	34.7	34.4	1.42		
d 105	35.8	33.3	1.40		
Average ^2^	35.3	33.6	1.33		
Hemoglobin (g/dL)				0.40	0.37
d 1	10.6	10.9	0.21		
d 45	12.3	11.4	0.20		
d 75	12.4	12.4	0.20		
d 105	12.7	12.1	0.21		
Average ^2^	12.4	11.9	0.20		
Leukocytes (×10^3^ µL)				0.02	0.01
d 1	7.07	6.88	1.01		
d 45	13.0	11.2	1.17		
d 75	8.16 ^a^	6.45 ^b^	1.00		
d 105	8.20 ^a^	6.28 ^b^	1.00		
Average ^2^	9.78 ^a^	7.97 ^b^	0.96		
Granulocyte (×10^3^ µL)				0.03	0.01
d 1	1.68	1.16	0.32		
d 45	2.86	2.61	0.30		
d 75	2.00	1.72	0.31		
d 105	2.66 ^a^	1.44 ^b^	0.30		
Average ^2^	2.50 ^a^	1.91 ^b^	0.25		
Lymphocytes (×10^3^ µL)				0.05	0.01
d 1	4.07	4.35	0.55		
d 45	8.16	7.03	0.64		
d 75	5.11 ^a^	3.41 ^b^	0.51		
d 105	4.38	3.77	0.51		
Average ^2^	5.88 ^a^	4.72 ^b^	0.48		
Monocytes (×10^3^ µL)				0.68	0.29
d 1	1.31	1.36	0.11		
d 45	1.98	1.57	0.11		
d 75	1.04	1.11	0.12		
d 105	1.15	1.06	0.13		
Average ^2^	1.39	1.24	0.11		
Platelets (×10^3^ µL)				0.06	0.02
d 1	309	337	2.41		
d 45	207 ^b^	240 ^a^	2.40		
d 75	199 ^b^	249 ^a^	2.40		
d 105	239	238	2.45		
Average ^2^	215	242	2.04		

^1^ Treatments were: Control (144 mg monensin/animal/day); and Treatment (552 mg curcumin/animal/day). ^2^ Significant treatment effect when *p* ≤ 0.05 illustrated by different letters on the same line (average); ^3^ Treatment × day interaction when *p* ≤ 0.05 illustrated by different letters on the same line on each data collection day. SEM: standard error.

**Table 4:** Serum biochemistry of cattle consuming of curcumin with additive.

Items	Treatments ^1^		SEM	*p*-Values	
	Control	Curcumin		Treat ^2^	Treat × Day ^3^
Albumin (g/dL)				0.82	0.75
d 1	4.21	4.57	0.36		
d 45	3.45	3.18	0.35		
d 75	3.13	3.21	0.30		
d 105	1.56	2.02	0.21		
Average ^2^	2.71	2.80	0.29		
Globulin (g/dL)				0.10	0.08
d 1	5.99	4.96	0.33		
d 45	3.45	4.62	0.30		
d 75	5.12	5.56	0.30		
d 105	10.3	11.0	0.38		
Average ^2^	6.29	7.06	0.27		
Total protein (g/dL)				0.14	0.22
d 1	10.2	9.53	0.41		
d 45	6.90	7.80	0.34		
d 75	8.25	8.77	0.37		
d 105	11.9	13.1	0.37		
Average ^2^	9.01	9.89	0.32		
Glucose (mg/dL)				0.04	0.01
d 1	151	147	3.08		
d 45	102	105	2.10		
d 75	82.7 ^a^	73.8 ^b^	1.94		
d 105	101 ^a^	83.7 ^b^	1.99		
Average ^2^	95.2 ^a^	87.5 ^b^	1.90		
Cholesterol (mg/dL)				0.01	0.01
d 1	83.6	88.1	2.63		
d 45	82.0	91.7	2.65		
d 75	70.2 ^b^	90.6 ^a^	2.97		
d 105	63.2 ^b^	71.8 ^a^	1.80		
Average ^2^	71.8 ^b^	84.7 ^a^	1.85		
Urea (mg/dL)				0.84	0.87
d 1	23.4	24.8	1.36		
d 45	19.7	22.6	1.29		
d 75	27.0	29.2	1.29		
d 105	24.3	23.3	1.25		
Average ^2^	23.6	25.0	1.23		

^1^ Treatments were: Control (144 mg monensin/animal/day); and Treatment (552 mg curcumin/animal/day). ^2^ Significant treatment effect when *p* ≤ 0.05 illustrated by different letters on the same line (average); ^3^ Treatment × day interaction when *p* ≤ 0.05 illustrated by different letters on the same line on each data collection day. SEM: standard error.

**Table 5:** Oxidative status of cattle consuming of curcumin with additive.

Items	Treatments ^1^		SEM	*p*-Values	
	Control	Curcumin		Treat ^2^	Treat × Day ^3^
TBARS (mmol MDA/mL)				0.03	0.01
d 1	13.8	13.2	0.94		
d 45	24.6 ^a^	10.4 ^b^	1.39		
d 75	20.1 ^a^	14.2 ^b^	0.97		
d 105	22.7 ^a^	14.1 ^b^	0.92		
Average ^2^	22.4 ^a^	12.9 ^b^	1.09		
Total Thiol (µmol/L)				0.01	0.01
d 1	79.4	73.8	2.10		
d 45	64.8 ^b^	76.9 ^a^	2.11		
d 75	62.8 ^b^	74.0 ^a^	2.09		
d 105	55.8 ^b^	75.2 ^a^	1.89		
Average ^2^	61.3 ^b^	75.3 ^a^	1.97		
GST (nmol/min/mg of protein)				0.05	0.10
d 1	333	313	6.78		
d 45	323	335	6.54		
d 75	335	352	6.65		
d 105	376	398	6.21		
Average ^2^	344 ^b^	361 ^a^	5.09		

^1^ Treatments were: Control (144 mg monensin/animal/day); and Treatment (552 mg curcumin/animal/day). ^2^ Significant treatment effect when *p* ≤ 0.05 illustrated by different letters on the same line (average); ^3^ Treatment × day interaction when *p* ≤ 0.05 illustrated by different letters on the same line on each data collection day. SEM: standard error.

**Table 6:** Ruminal fluid of cattle consuming of curcumin with additive.

Items	Treatments ^1^		SEM ^5^	*p*-Values	
	Control	Curcumin		Treat ^2^	Treat × Day ^3^
pH	6.64	6.67	0.02	0.94	0.40
MBRT ^4^ (second)				0.05	0.03
d 75	119 ^a^	78.7 ^b^	5.62		
d 105	79.5	62.7	3.74		
Average ^2^	99.2 ^a^	70.7 ^b^	3.24		
Protozoa number (×10^5^/mL)				0.04	0.01
d 75	11.2 ^b^	15.4 ^a^	0.75		
d 105	10.5	12.5	0.75		
Average ^2^	10.8 ^b^	13.9 ^a^	0.70		
Total short-fatty acid (nmol/L)	102	100	0.60	0.78	0.18
Acetic acid (%)	67.8	68.4	1.25	0.89	0.92
Propionic acid (%)	17.6	17.7	0.54	0.92	0.94
Isobutyric acid (%)	0.81	0.74	0.03	0.21	0.15
Butyric acid (%)	11.0	10.7	0.93	0.91	0.86
Isovaleric acid (%)	1.39	1.16	0.12	0.22	0.11
Valeric acid (%)	1.23	1.21	0.09	0.95	0.92

^1^ Treatments were: Control (144 mg monensin/animal/day); and Treatment (552 mg curcumin 100/animal/day). ^2^ Significant treatment effect when *p* ≤ 0.05 illustrated by different letters on the same line (average); ^3^ Treatment × day interaction when *p* ≤ 0.05 illustrated by different letters on the same line on each data collection day. ^4^ Methylene blue reduction test (MBRT); ^5^ SEM: standard error.

**Table 7:** Results post-mortem of cattle consuming of curcumin with additive: Carcass and meat.

Variables	Control ^1^	Curcumin ^1^	SEM	*p*-Value ^2^
Carcass yield (%)	50.8	50.3	0.65	0.95
pH	5.45	5.48	0.02	0.92
Water retention capacity (%)	78.2 ^b^	82.0 ^a^	1.02	0.05
Lightness	39.2	40.8	0.35	0.90
Color a (redness)	15.5	16.9	0.64	0.36
Color b (yellowness)	12.2 ^b^	14.5 ^a^	0.53	0.05
Moisture (%)	71.4	71.4	0.12	0.97
Protein (%)	22.7	23.2	0.20	0.84
Total lipids (%)	2.10	2.08	0.03	0.75
TBARS (mmol MDA/mg protein)	12.5 ^a^	8.74 ^b^	1.13	0.01
SOD (U SOD/mg protein)	41.6 ^b^	63.7 ^a^	3.74	0.01

^1^ Treatments were: Control (144 mg monensin/animal/day); and Treatment (552 mg curcumin/animal/day). ^2^ Significant treatment effect when *p* ≤ 0.05 illustrated by different letters on the same line (average). SEM: standard error.

**Table 8:** Fatty acid profile of cattle consuming of curcumin with additive.

Fatty Acid (%)	Control ^1^	Curcumin ^1^	SEM	*p*-Value ^2^
C12:0 (Lauric)	0.040	0.043	0.001	0.814
C14:0 (Myristic)	1.798	0.736	0.025	0.001
C14:1 (Myristoleic)	0.413	0.351	0.027	0.369
C15:0 (Pentadecanoic)	0.213	0.201	0.018	0.874
C16:0 (Palmitic)	28.7 ^a^	26.1 ^b^	0.084	0.003
C16:1 (Palmitoleic)	3.077	2.943	0.041	0.751
C17:0 (Heptadecanoic)	0.708	0.666	0.029	0.732
C18:0 (Stearic)	15.803	16.953	0.084	0.067
C18:1n9c (Oleic)	39.7 ^b^	42.1 ^a^	0.102	0.025
C18:2n6c (Linoleic)	6.059	6.686	0.049	0.504
C20:0 (Arachidic)	0.137	0.151	0.008	0.142
C18:3n6 (Linolenic)	0.080 ^a^	0.067 ^b^	0.002	0.004
C20:1n9 (cis-11-Eicosenoic)	0.205	0.200	0.009	0.914
C18:3n3 (a-Linolenic)	0.207	0.175	0.013	0.195
C20:2 (cis-11,14-Eicosadienoic)	0.088	0.086	0.005	0.954
C22:0 (Behenic)	0.068	0.071	0.004	0.895
C20:3n6 (cis-8,11,14-Eicosatrienoic)	0.478	0.469	0.023	0.920
C22:1n9 (Erucic)	0.033 ^b^	0.062 ^a^	0.006	0.002
C20:4n6 (Arachidonic)	1.905	1.683	0.036	0.083
C22:2 (cis-13,16-Docosadienoic)	0.016	0.017	0.008	0.961
C24:0 (Lignoceric)	0.065	0.071	0.005	0.794
C20:5n3 (cis-5,8,11,14,17-Eicosapentaenoic)	0.043 ^a^	0.000 ^b^	0.002	0.009
C24:1n9 (Nervonic)	0.031	0.033	0.008	0.957
C22:6n3 (cis-4,7,10,13,16,19-Docosahexaenoic)	0.029	0.038	0.007	0.251
SFA—saturated fatty acids	47.5 ^a^	45.0 ^b^	0.143	0.021
MUFA—monosaturated fatty acids	43.5 ^b^	45.6 ^a^	0.129	0.016
PUFA—polyunsaturated fatty acids	8.817	9.136	0.082	0.574
UFA—unsaturated fatty acids	52.34	54.82	0.135	0.005

^1^ Treatments were: Control (144 mg monensin/animal/day); and Treatment (552 mg curcumin/animal/day). ^2^ Significant treatment effect when *p* ≤ 0.05 illustrated by different letters on the same line (average). SEM: standard error.

## Data Availability

Data will be made available upon request.

## Supplementary Materials

The following supporting information can be downloaded at: https://www.mdpi.com/article/10.3390/ani16020174/s1, Table S1: Standardization of volatile fatty acid quantification in rumen fluid; Table S2: Fatty acid profile in feed consumed by cattle.
