# Supplementary material for: Curcumin as a Dietary Additive in Early-Finished Feedlot Steers and Its Effects on Performance, Ruminal Environment, Animal Health, and Meat Quality
Source: Animals (Basel). 2026 Jan 7;16(2):174. doi: 10.3390/ani16020174 (PMC12837996; doi:10.3390/ani16020174)
Supplement: Supplementary file 1 [file animals-16-00174-s001.zip › animals-4072843-supplementary.pdf]

Table S1: Standardization of volatile fatty acid quantification in rumen fluid.

	Acetic acid	Propionic acid	Butiric acid	Isovaleric acid	Valeric acid
**R^2**	0.9963	0.9982	0.9983	0.9985	0.9996
**Equation**	y = 0.0097x + 0.0279	y = 0.0179x + 0.0007	y = 0.0247x + 0.0005	y = 0.0318x + 0.0018	y = 0.0297x – 0.0005
**Linear range (mmol L^{-1})***	4.28 – 128.37	1.76 – 70.51	1.31 – 52.24	0.27 – 13.26	0.28 – 13.55
**LOD (mmol L^{-1})**	1.07	0.88	0.65	0.27	0.28
**LOQ (mmol L^{-1})**	2.14	1.76	1.31	0.55	0.56
**Accuracy (%)**	107.67	110.78	108.41	103.82	113,48
**Repeatability (RSD)**	1.33	0.59	0.38	1.50	0.44

The linear range, LOD (limit of detection) and LOQ (limit of quantitation) were expressed in mmol of SFA for L of ruminal fluid.

Table S2: Fatty acid profile in feed consumed by cattle

Fatty acid	Concentrate monensin	Concentrate curcumin	Silage
C6:0 (Caproic)	0.096	0.092	0.000
C8:0 (Caprylic)	0.022	0.022	0.000
C12:0 (Lauric)	0.017	0.020	0.234
C14:0 (Myristic)	0.131	0.151	0.453
C15:0 (Pentadecanoic)	0.099	0.102	0.107
C16:0 (Palmitic)	24.54	23.90	21.92
C16:1 (Palmitoleic)	0.256	0.302	0.279
C17:0 (Heptadecanoic)	0.198	0.231	0.493
C17:1 (cis-10-Heptadecenoic)	0.000	0.000	0.029
C18:0 (Stearic)	5.194	4.688	6.485
C18:1n9t (Elaidic)	1.036	0.974	0.467
C18:1n9c (Oleic)	30.72	31.13	28.09
C18:2n6t (Linolelaidic)	0.000	0.000	0.138
C18:2n6c (Linoleic)	33.60	34.26	33.54
C20:0 (Arachidic)	0.750	0.697	0.806
C20:1n9 (cis-11-Eicosenoic)	0.523	0.534	0.252
C18:3n3 (a-Linolenic)	1.470	1.500	4.774
C22:0 (Behenic)	0.501	0.457	0.759
C22:1n9 (Erucic)	0.141	0.198	0.061
C20:3n3 (cis-11.14.17-Eicosatrienoic)	0.147	0.135	0.214
C20:4n6 (Arachidonic)	0.000	0.059	0.000
C22:2 (cis-13.16-Docosadienoic)	0.000	0.479	0.886
C24:0 (Lignoceric)	0.548	0.049	0.000
